# Ethanol Extract of *Limonium bicolor* Improves Dextran Sulfate Sodium-Induced Ulcerative Colitis by Alleviating Inflammation and Restoring Gut Microbiota Dysbiosis in Mice

**DOI:** 10.3390/md22040175

**Published:** 2024-04-15

**Authors:** Wei Jia, Siyu Yu, Xi Liu, Qingqing Le, Xiwen He, Lutao Yu, Jianlin He, Longhe Yang, Huiyuan Gao

**Affiliations:** 1 School of Traditional Chinese Materia Medica, Shenyang Pharmaceutical University, Shenyang 110016, China; jiawei20210226@163.com (W.J.); ysylogin@163.com (S.Y.); a1907826062@163.com (L.Y.); 2 Technical Innovation Center for Utilization of Marine Biological Resources, Third Institute of Oceanography, Ministry of Natural Resources, Xiamen 361000, China; leqingqing@tio.org.cn (Q.L.); hexiwen1224@163.com (X.H.); jlhe@tio.org.cn (J.H.); 3 Key Laboratory of Chemical Biology of Fujian Province, Department of Chemical Biology, College of Chemistry and Chemical Engineering, Xiamen University, Xiamen 361000, China; lancyliu2584@163.com; 4 Fujian Key Laboratory of Island Monitoring and Ecological Development (Island Research Center, Ministry of Natural Resources), Fuzhou 350400, China

**Keywords:** *Limonium bicolor*, ulcerative colitis, gut microbiota, inflammation, RAW 264.7 cell

## Abstract

Ulcerative colitis (UC) is a kind of inflammatory bowel condition characterized by inflammation within the mucous membrane, rectal bleeding, diarrhea, and pain experienced in the abdominal region. Existing medications for UC have limited treatment efficacy and primarily focus on symptom relief. Limonium bicolor (LB), an aquatic traditional Chinese medicine (TCM), exerts multi-targeted therapeutic effects with few side effects and is used to treat anemia and hemostasis. Nevertheless, the impact of LB on UC and its mechanism of action remain unclear. Therefore, the objective of this study was to investigate the anti-inflammatory effects and mechanism of action of ethanol extract of LB (LBE) in lipopolysaccharide-induced RAW 264.7 macrophages and dextran sulfate sodium (DSS)-induced UC. The results showed that LBE suppressed the secretion of cytokines in LPS-stimulated RAW 264.7 cells in a dose-dependent manner. LBE had protective effects against DSS-induced colitis in mice, decreased the disease activity index (DAI) score, alleviated symptoms, increased colon length, and improved histological characteristics, thus having protective effects against DSS-induced colitis in mice. In addition, it reversed disturbances in the abundance of proteobacteria and probiotics such as *Lactobacillus* and *Blautia* in mice with DSS-induced UC. Based on the results of network pharmacology analysis, we identified four main compounds in LBE that are associated with five inflammatory genes (*Ptgs2*, *Plg*, *Ppar-γ*, *F2*, and *Gpr35*). These results improve comprehension of the biological activity and functionality of LB and may facilitate the development of LB-based compounds for the treatment of UC.

## 1. Introduction

Ulcerative colitis (UC) is classified as an inflammatory bowel disease that causes damage to the colon and rectum. Although the precise etiology of UC remains elusive, several factors have been reported to contribute to its pathogenesis, including dysregulated immune responses, gut microbiota dysbiosis, genetic predisposition, and environmental stimuli [1]. In the clinical context, UC is characterized by superficial inflammation of the colonic mucous membrane, bleeding from the rectum, occurrences of diarrhea, and sensations of abdominal discomfort [2]. These conditions disrupt the normal dietary intake and digestive function of patients, consequently diminishing their quality of life. Histologically, UC is characterized by extensive crypt architectural distortion, transmucosal inflammatory infiltration with basal plasmacytosis, cryptitis, and crypt abscesses [3]. During the development of UC, activated macrophages generate abundant cytokines (e.g., tumor necrosis factor-alpha [TNF-α] and interleukin [IL]-6) along with reactive metabolites of oxygen and nitrogen [4]. Dysbiosis of intestinal microbiota is an important factor in UC. Microbial composition shapes the colonic environment, as the metabolic products of microbes may participate in signaling, modulate the immune system, or exhibit antibiotic activity [5]. 

At present, UC is primarily managed with symptomatic treatment, with established drugs such as 5-aminosalicylic acid (5-ASA), thiopurines, anti-TNF (TNFi) agents, and vedolizumab being used for the maintenance of remission [6]. However, these drugs have several limitations; for instance, steroids are unsuitable for long-term use owing to their adverse effects and inability to maintain remission, whereas azathioprine has limited treatment efficacy in acute settings [7]. 

Marine traditional Chinese medicine (TCM) hosts a vast reservoir of medical resources. Its multi-targeted mode of action and minimal side effects offer unique advantages in managing chronic diseases, including definitive therapeutic effects, effective maintenance, reduced recurrence rates, and fewer toxic effects [8]. Consequently, TCM holds potential as a complementary medicinal practice in the treatment of UC.

*Limonium bicolor* (LB) is indigenous to saline–alkaline environments along coastlines and inland regions such as plains, hills, or sandy lands [9]. It has been widely used in TCM owing to its efficacy in treating anemia, promoting hemostasis, alleviating menstrual disorders, and combating uterine carcinoma [10]. The literature reviews have reported the presence of polysaccharides, flavonoids, steroids, and sulfated phenolics in *Limonium* species [10]. However, studies investigating the therapeutic potential of LB in UC are lacking. 

In this study, the ethanol extract of LB (LBE) was found to alleviate UC in vivo and in vitro. In particular, LBE reduced the production of nitrite and TNF-α in lipopolysaccharide (LPS)-stimulated RAW 264.7 cells in vitro in a dose-dependent manner and effectively ameliorated dextran sodium sulfate (DSS)-induced UC in vivo. LC-MS/MS revealed 15 representative compounds in LBE. Network pharmacological analysis showed that LBE exerted protective effects against UC through five genes, namely, *Ptgs2*, *Plg*, *Ppar-γ*, *F2*, and *Gpr35*. Finally, 16S rRNA sequencing of fecal samples suggested that LBE protected against UC by altering the configuration of gut microbiota, reducing the abundance of opportunistic pathogens, and promoting the growth of beneficial bacteria in the gut microbiota.

## 2. Results

### 2.1. LBE Inhibited the Expression of Pro-Inflammatory Cytokines in LPS-Stimulated RAW264.7 Cells

**Figure 1:**
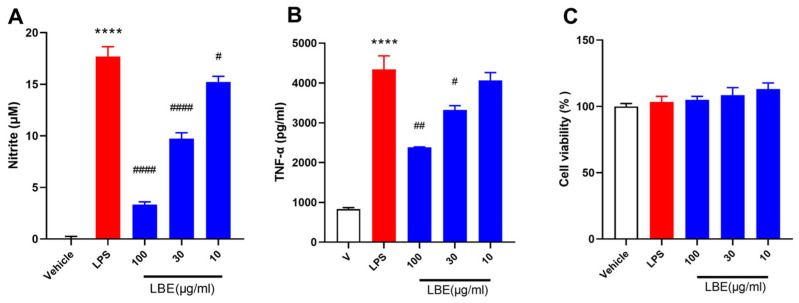
Effects of LBE on nitrite production (**A**), TNF-α production (**B**), and cell viability (**C**) in LPS-stimulated RAW 264.7 cells. All data are expressed as the mean ± SEM, n = 3. Significant differences were analyzed via one-way ANOVA with Tukey’s multiple comparison test (****, *p* < 0.0001 for the LPS versus vehicle group; ^#^, *p* < 0.05; ^##^, *p* < 0.01; ^####^, *p* < 0.0001 for the LPS + LBE versus DSS group).

The anti-inflammatory effects of LBE- on LPS-induced RAW264.7 cells were investigated by assessing the levels of nitrite and TNF-α in vitro. As shown in Figure 1A, LBE inhibited the production of nitrite at a concentration of 10–100 μg/mL in a dose-dependent manner and effectively attenuated the LPS-induced increase in TNF-α levels at a concentration of 30 and 100 μg/mL (Figure 1B). In addition, LBE did not have cytotoxic effects at any tested concentration, including the highest concentration of 100 μg/mL. These findings indicated that LBE maintained the viability of RAW264.7 cells while inhibiting the LPS-induced inflammatory effects.

### 2.2. LBE Attenuated DSS-Induced Colon Tissue Damage and Decreased DAI Scores

**Figure 2:**
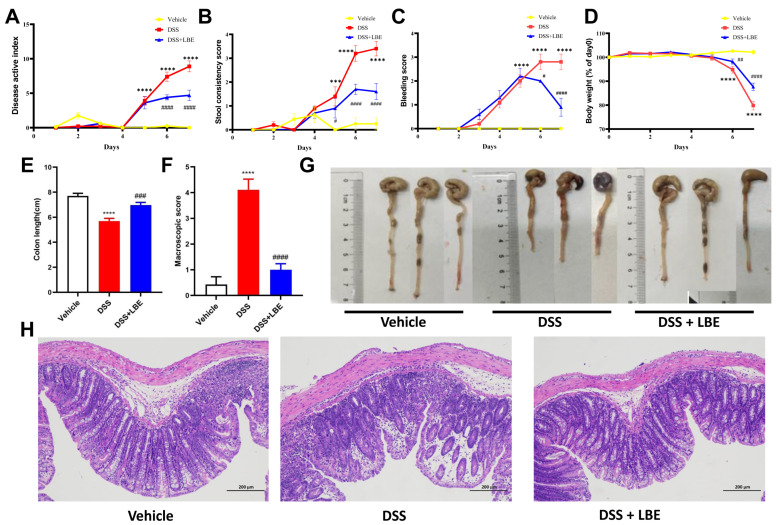
Disease active index score (**A**), stool consistency score (**B**), bleeding score (**C**), body weight score (**D**), colon length and representative images (**E**,**G**), macroscopic scores (**F**), and results of histochemical analysis (**H**). All data are expressed as the mean ± SEM, n = 6–8. Significant differences were analyzed using one-way ANOVA with Tukey’s multiple comparison test (*** *p* < 0.001, **** *p* < 0.0001 for the DSS versus vehicle group; ^#^ *p* < 0.05; ^##^, *p* < 0.01; ^###^ *p* < 0.001; and ^#####^ *p* < 0.0001 for the DSS + LBE versus DSS group).

For the purpose of examining the therapeutic efficacy of LBE in relation to UC in an in vivo context, C57BL6/J mice were orally administered 3% DSS in their drinking water over a period of 7 days to elicit acute colitis [11]. Based on the estimation of the effective dose of LBE through in vitro experiments, we selected 30 mg/kg as the optimal dose for animal experiments. As shown in Figure 2A, DAI scores were lower in the LBE group than in the model group, suggesting that administration of LBE significantly suppressed the development of DSS-induced colitis. Mice treated with DSS experienced weight loss when compared with those treated with a vehicle; however, treatment with LBE reversed the DSS-induced weight loss (Figure 2D). In addition, treatment with LBE significantly alleviated DSS-induced diarrhea (Figure 2B) and bleeding (Figure 1C). The beneficial effects of LBE on UC were further validated in mouse colon tissue samples. As shown in Figure 1E–G, LBE partially prevented the DSS-induced shortening of the colon. 

Histochemical analysis revealed crypt structural alterations characterized by erosive and ulcerative lesions in the colons of mice with DSS-induced UC (Figure 2H). Inflammatory infiltrates were observed in the epithelial layer surrounding the lesions. Additionally, it decreased the number of crypt goblet cells, the size of vacuoles in goblet cells was decreased, and myofibroblasts around crypts were partially or completely depleted (Figure 2H). However, treatment with LBE partially reversed the aforementioned DSS-induced pathological changes in the mouse colon.

### 2.3. LBE Decreased the Levels of Pro-Inflammatory Cytokines

**Figure 3:**
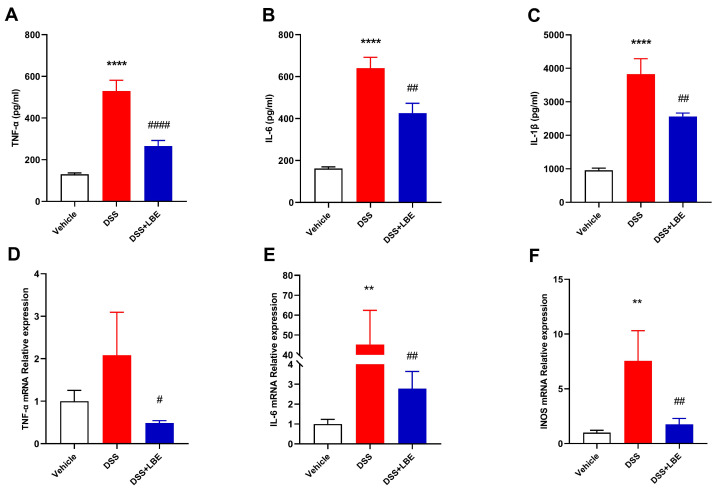
Serum levels of TNF-α (**A**), IL-6 (**B**), and IL-1β (**C**). mRNA expression of TNF-α (**D**), IL-6 (**E**), and iNOS (**F**). Data are presented as the fold change using GAPDH as the internal reference (** *p* < 0.01; **** *p* < 0.0001 for the DSS versus vehicle group; ^#^ *p* < 0.05; ^##^ *p* < 0.01; ^####^ *p* < 0.0001 for the DSS + LBE versus DSS group). Differences were analyzed using one-way ANOVA with Tukey’s multiple comparison test (mean ± SEM, n = 6–8).

Changes in the levels of pro-inflammatory cytokines, such as TNF-α, IL-6, and IL-1β, in serum and the mRNA expression of pro-inflammatory factors, such as TNF-α, IL-6, and iNOS, in the colon are associated with both exacerbation and alleviation of inflammation. Consistent with the symptoms of DSS-induced UC and the results of histochemical analysis, the levels of TNF-α, IL-6, and IL-1β in serum were significantly higher in DSS-treated mice than in vehicle-treated mice. However, treatment with LBE decreased the levels of these pro-inflammatory cytokines in the serum of DSS-treated mice (Figure 3A–C). Furthermore, DSS markedly increased the mRNA expression of TNF-α, IL-6, and iNOS in the mouse colon, whereas LBE reversed this effect (Figure 3D–F).

### 2.4. Identification of the Chemical Components of LBE

**Figure 4:**
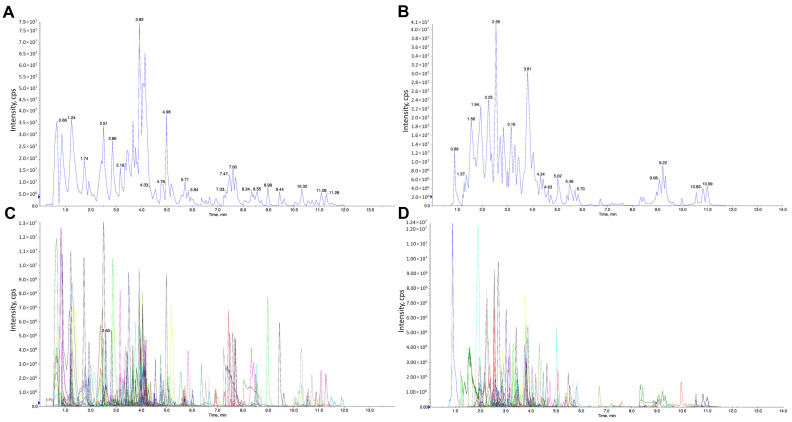
Total ion chromatogram in the positive mode (**A**) and negative mode (**B**) in MRM. Extracted ion chromatogram in the positive mode (**C**) and negative mode (**D**) in MRM.

The chemical components of LBE were identified via LC-MS/MS. Figure 4A,B show the total extracted ion chromatogram (TIC, that is, the plot in which the intensities of all ions in the mass spectrum at each time point are summed against time). Figure 4C,D show the multimodal plot of metabolites identified via MRM (the ion current spectrum of the multi-substance extraction, Xic), with the abscissa representing the retention time (RT) for metabolite detection and the ordinate representing the ion current intensity (in count per second [CPS]) for ion detection. Each distinct colored mass spectral peak in the MRM plot represents one identified component. The characteristic ions of the identified components were screened using a triple quaternary rod, the signal intensity of the ions was obtained in a detector, and the chromatographic peaks and correction were integrated, with the area of each peak representing the relative content of the corresponding component. The components of LBE screened via MS were identified and quantified using the MetWare database (a metabolite database). Finally, 15 major chemical components were identified (Table 1), including gallotannins, N-benzylmethylene isomethylamine, N-benzoyl-2-aminoethyl-β-D-glucopyranoside, gallic acid, and flavonoids.

### 2.5. Potential Targets of LBE Predicted via Network Pharmacological Analysis

**Figure 5:**
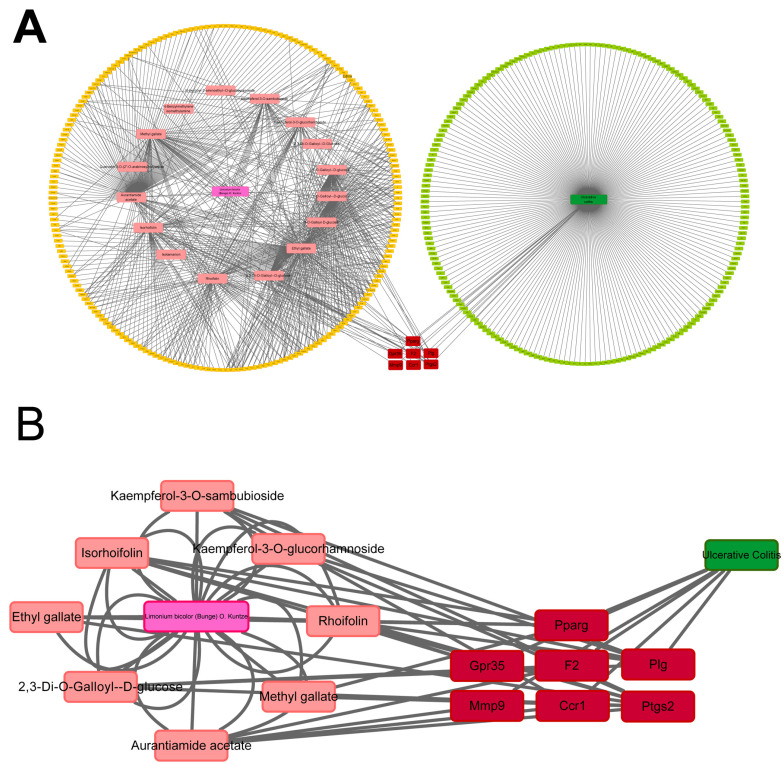
Chemical compound–target–disease network. (**A**) Overview. (**B**) Partial view.

**Figure 6:**
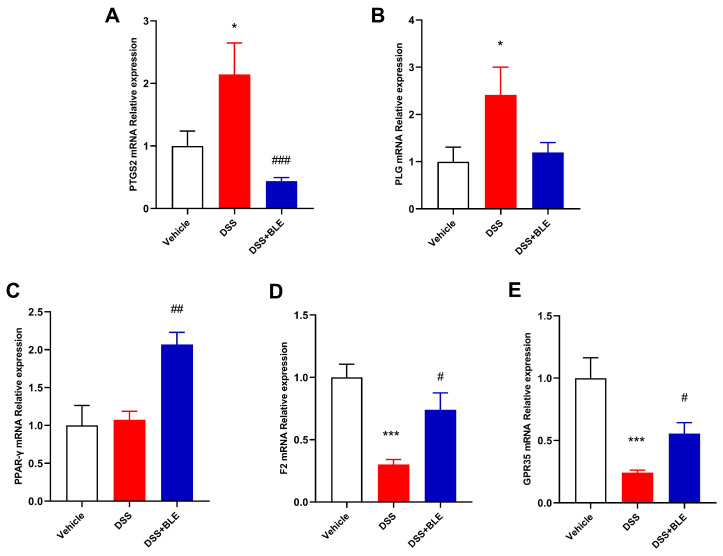
Relative mRNA expression of LBE–UC common genes. (**A**) PTGS2. (**B**) PLG. (**C**) PPAR-γ. (**D**) F2. (**E**) GPR35. Data are expressed as the mean ± SEM (n = 6–8) (* *p* < 0.05; *** *p* < 0.001, DSS versus vehicle group; ^#^ *p* < 0.05; ^##^ *p* < 0.01; ^###^ *p* < 0.001, DSS + LBE versus DSS group).

Network pharmacological analysis was performed to predict the potential targets of the 15 compounds of LBE. Initially, the Swiss Target Prediction Database was used to predict and summarize the corresponding targets of the 15 compounds. Simultaneously, the GeneCards database was used to identify therapeutic targets for UC based on the cutoff score of >10. Subsequently, the Cytoscape software was used to visualize a chemical compound–target–disease network constructed based on the 15 chemical components of LBE, the corresponding targets of the 15 components, and UC-related targets (Figure 5A,B). The common targets and the degree scores calculated based on the number of intersections are shown in Table 2. Based on their higher scores, five genes were identified as putative targets of LBE in the therapeutic treatment of UC. Real-time PCR was performed to validate whether these genes served as the targets of LBE. The results showed that LBE reversed the effects of DSS on the expression of *Ptsg2*, *Plg*, *Ppar-γ*, *F2*, and *Gpr35* (Figure 6). These preliminary findings suggest that LBE exerts protective effects against UC through *Ptsg2*, *Plg*, *Ppar-γ*, *F2*, and *Gpr35*. 

### 2.6. Effects of LBE on the Gut Microbiota of Mice with DSS-Induced UC

**Figure 7:**
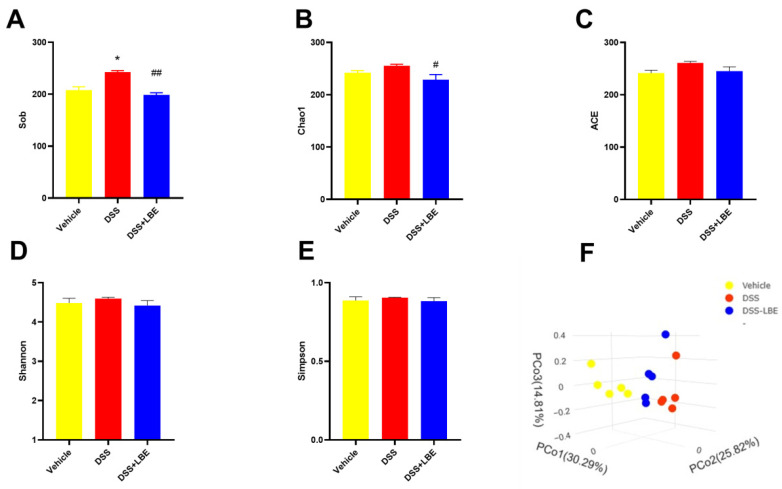
Gut microbial diversity and abundance in the vehicle, DSS, and DSS-LBE groups. (**A**) Sob index. (**B**) Chao1 index. (**C**) ACE index. (**D**) Shannon index. (**E**) Simpson index. (**F**) PCoA. Data are expressed as the mean ± SEM, n = 5. (* *p* < 0.05, DSS versus vehicle group; ^#^ *p* < 0.05; ^##^ *p* < 0.01, DSS + LBE versus DSS group).

Given that the major active components of LBE were found to be flavonoids, which are difficult to dissolve in water, we speculated that LBE protected mice against UC through the gut microbiota. To verify this hypothesis, we assessed the composition of gut microbiota in mice through 16S rRNA sequencing and evaluated the abundance and diversity of gut microbial species using α diversity indices. The Sob, Chao1, and ACE indices reflect species abundance, whereas the Shannon and Simpson indices reflect species diversity. The results showed that administration of DSS increased species abundance in mice, whereas administration of LBE reversed this effect (Figure 7A–C). On the contrary, species diversity was influenced by neither DSS nor LBE (Figure 7D–E). These results indicated that DSS-induced UC damaged the microenvironment of the colon, leading to the rapid growth of opportunistic pathogens, which contributed to the elevated species abundance. Furthermore, principal coordinate analysis (PCoA), a method for dimensionality reduction, was used to assess β diversity, which reflects the connection and discrimination between bacterial communities. As shown in the scatterplot in Figure 7F, significant differences were observed in the structure of gut microbiota between the vehicle and DSS groups. The structure of gut microbiota in LBE-treated mice was more similar to that in vehicle-treated mice than that in DSS-treated mice, suggesting that LBE has the capacity to reconstitute the configuration of the intestinal microbiome in mice afflicted with DSS-induced UC.

**Figure 8:**
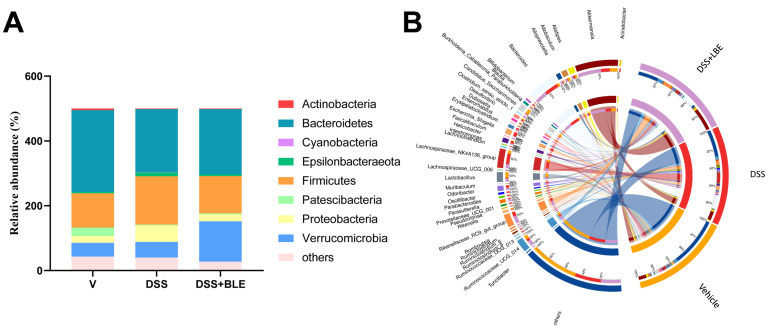
Species composition at the genus level. (**A**) Stacking diagram of dominant phyla. (**B**) Circle diagram of dominant genera.

To compare species composition among groups, we classified 15 phyla and 134 genera based on 99% of the total bacterial abundance. Bacteria with a relative abundance of >1% in each group were considered dominant. Subsequently, DSS- and LBE-related bacteria were identified. As shown in Figure 8A, the relative abundance of *p_Proteobacteria* and *p_Epsilonbacteraeota*, which are pathogenic bacteria, was higher in the DSS group than in the DSS + LBE group. The ratio of *p_Firmicutes* to *p_Bacteroidetes* (F/B) indicates the balance of gut microbiota. An increased F/B ratio is observed in most conditions characterized by intestinal inflammation [12]. In this study, the F/B ratio was 1.83 times higher in the colon of mice with DSS-induced UC than in the colon of vehicle-treated mice, whereas administration of LBE significantly restored the F/B ratio in mice with UC. Among the dominant genera, 11 genera had markedly differential abundance across groups (Figure 8B). The abundance of *g_Candidatus_saccharimonas*, *g_Lactobacillus*, and *g_Akkermansia*, which are probiotics, was lower in the DSS group than in the LBE group. The abundance of *g_Blautia*, *g_Lachnospiraceae_UCG-006*, *g_Ruminiclostridium_9*, *g_Oscillibacter*, and *g_Erysipelatoclostridium* was increased in the DSS group. However, treatment with LBE decreased the relative abundance of *g_Blautia*, *g_Lachnospiraceae_UCG-006*, *g_Ruminiclostridium_9*, and *g_Oscillibacter* in mice with UC. *g_Blautia*, *g_Lachnospiraceae_UCG-006*, and *g_Ruminiclostridium_9* have been associated with the development of IBD and chronic inflammation. Altogether, these results indicate that LBE protects against UC by regulating the composition of gut microbial species and promoting the reproduction of probiotics in the intestine.

## 3. Discussion

In this study, we investigated the protective effects of LBE against DSS-induced UC in mice and integrated network pharmacological analysis with 16S rRNA sequencing to elucidate the mechanism of action of LBE. In UC, dysregulation of the immune system promotes the secretion of pro-inflammatory cytokines, such as TNF-α, IL-6, and IL-1β, which in turn promote the activation and proliferation of lymphocytes [13,14]. Increased mRNA expression of TNF-α, IL-6, and IL-1β has been observed on colon mucosal biopsy in patients with UC [15]. In this study, LBE suppressed the production of nitrite and pro-inflammatory factors in LPS-stimulated RAW 264.7 cells in a dose-dependent manner in vitro. In addition, LBE decreased the DAI score and the levels of pro-inflammatory factors in the serum and colon of mice with DSS-induced UC. These findings indicate that LBE exerts anti-inflammatory effects against UC.

To examine the mechanism of action of LBE in the treatment of UC, a compound–target–disease network was constructed to visualize the intersection between potential targets of LBE- and UC-related genes. Eventually, five common targets with a high score of intersection were identified, namely, *Ptgs2*, *F2*, *Plg*, *Pparγ*, and *Gpr35*. On the one hand, the mRNA expression of *Ptgs2* and *Plg* was higher in the DSS group than in the DSS + LBE group. On the other hand, the mRNA expression of *F2* and *Gpr35* was lower in the DSS group than in the DSS + LBE group. Based on the results of network pharmacological analysis, isotamarixin and quercetin-3-O-(2″-O-arabinosyl) rutinoside are the potential active components of LBE that target *Ptgs2*, *Plg*, *F2*, and *Gpr35*, whereas isotamarixin, quercetin-3-O-(2″-O-arabinosyl) rutinoside, isorhoifolin, and rhoifolin may target only *Ptgs2*, *Plg*, and *Gpr35*.

The *Ptgs2* gene encodes COX-2, which is a type of inducible synthase. COX-2 is expressed in response to inflammation and physical stress and participates in the mediation of pain and the production of prostaglandin [16]. Notably, rhoifolin, which is also found in *Citrus aurantium* L. var. amara Engl., has been shown to decrease the expression of COX-2 [17]. To the best of our knowledge, this study is the first to demonstrate the regulatory effects of isotamarixin, quercetin-3-O-(2″-O-arabinosyl) rutinoside, and isorhoifolin on the mRNA expression of *Ptgs2*. The *Plg* gene encodes plasminogen (PLG), the precursor of plasmin, and is activated by tissue plasminogen activator (tPA) and urokinase plasminogen activator (uPA). Activated PLG exerts anti-thrombotic effects by decomposing fibrin; however, excessive activation of PLG may increase the risk of bleeding [18]. F2, the precursor of thrombin, promotes the production of fibrous proteins, and its effects are contradictory to those of PLG. Inhibiting the expression of F2 can impede wound healing and increase the risk of bleeding [19]. Therefore, maintaining the balance between PLG and F2 may help alleviate hematochezia in DSS-induced UC. In this study, treatment with DSS increased the PLG/F2 ratio, whereas treatment with LBE counteracted this effect by increasing F2 levels and decreasing Plg levels. The effects of LBE and its components on the PLG/F2 ratio have been rarely reported. Based on the results of network pharmacological analysis and validation of mRNA expression via qRT-PCR, we speculate that isotamarixin and quercetin-3-O-(2″-O-arabinosyl) rutinoside are the potential active compounds of LBE that regulate the PLG/F2 ratio. GPR35, a G protein-coupled receptor (GPCR), is primarily expressed in the gastrointestinal tract and plays a key role in regulating gastrointestinal homeostasis. It is activated by endogenous metabolites such as lysophosphatidic acid, CXCL17 chemokine, and 5-hydroxyindoleacetic acid [20]. However, it is considered an orphan receptor, as its endogenous activator remains unknown. Deficiency of GPR35 has been associated with IBD, both UC and Crohn’s disease, as well as primary sclerosing cholangitis [21,22,23]. Electrolyte imbalance is usually the main cause of diarrhea in UC [24,25]. A recent study indicated that GPR35 might alleviate diarrhea by regulating iron homeostasis in UC [26]. In the present study, treatment with LBE reversed the inhibitory effects of DSS on Gpr35 expression. In addition, network pharmacological analysis and validation of mRNA expression via qRT-PCR indicated that isotamarixin, quercetin-3-O-(2″-O-arabinosyl) rutinoside, isorhoifolin, and rhoifolin might regulated the expression of Gpr35. The gene expression data presented in this study suggest potential targets for the therapeutic effects of the ethanol extract of Limonium bicolor. However, it is important to note that these findings indicate a direction for the extract’s mechanism of action rather than providing conclusive evidence at the protein level.

Furthermore, LC-MS/MS analysis revealed 15 components in LBE. Most components were classified as flavonoids, which exhibit multiple biological activities but have low bioavailability. Gut microbes can metabolize the part of flavonoids that cannot be absorbed by the blood, enhance the effects of drugs, and mediate intestinal bacterial homeostasis [27,28]. Therefore, we assessed the diversity of gut microbiota through 16S rRNA sequencing to understand the effects of LBE on gut microbiota in UC. The results showed that LBE increased the relative abundance of *g_Akkermansia* and *g_Lactobacillus* and decreased the relative abundance of *g_Blautia*, *g_Lachnospiraceae_UCG-006*, *g_Oscillibacter*, *g_Erysipelatoclostridium*, and *g_Ruminiclostridium_9*, thereby alleviating gut microbiota dysbiosis in mice with UC.

*g_Akkermansia*, a Gram-negative and strictly anaerobic bacterium belonging to the phylum *p_Verrucomicrobia*, usually colonizes the nutrient-rich intestinal mucosal layer [29,30]. It produces various endogenous metabolites (such as short-chain fatty acids) in the intestine to modulate host biological processes, such as glucose and lipid metabolism, and maintain gut barrier function through interactions between intestinal microbes and the host [31,32]. A recent study showed that *g_Akkermansia* may be closely related to the integrity of the intestinal mucosal barrier owing to its markedly decreased abundance in patients with IBD [33]. Increasing the abundance of *g_Akkermansia* can decrease the DAI score, restore mucosal architecture, and inhibit the release of pro-inflammatory cytokines in mice with DSS-induced UC, suggesting that *g_Akkermansia* holds substantial promise as a novel probiotic [34,35]. As one of the most well-known probiotics, *g_Lactobacillus* protects intestinal barrier function and mucosal immunity by increasing the thickness of the colonic mucosa [36,37,38,39]. In addition, it can alleviate UC by increasing the expression of TLR4/NF-κB, IL-22, and other immune-related genes, restoring intestinal mucosal barrier function, modulating gut microbiota dysbiosis, and inhibiting the growth of pathogenic bacteria [40,41,42].

*p_Proteobacteria* is predominantly found in the pathological states of endotoxemia and persistent inflammation, which are considered markers of microbial instability [43], especially in the intestinal tract of rats with UC [44,45]. Adhesion of microbes to intestinal epithelial cells (ECs) is key to inducing Th17 cells. *p_Proteobacteria* can adhere to the intestinal epithelium and promote the release of IFN-γ by inducing Th1 cells under the recognition of TLR9 [38,46]. This phenomenon may be one of the reasons for the imbalance of cytokines in UC. In this study, the levels of pro-inflammatory cytokines, namely, TNF-α, IL-6, and IL-10, and the relative abundance of *p_Proteobacteria* were increased in mice with DSS-induced UC. However, administration of LBE decreased the levels of the aforementioned pro-inflammatory cytokines in mice with UC. Although the expression of IFN-γ was not measured in this study, the changes observed in the cytokines tested in this study support the abovementioned phenomenon. Furthermore, the abundance of *g_Blautia* and *g_Oscillibacter* was significantly different between the DSS and DSS + LBE groups. *g_Blautia* is one of the dominant genera in the intestinal tract and is involved in metabolic disorders, inflammatory diseases, and biotransformation [47]. It is usually considered a probiotic; however, in this study, its abundance was higher in the DSS group than in the DSS + LBE group. Consistently, the abundance of *g_Blautia* has been reported to be higher in patients with irritable bowel syndrome and UC than in healthy individuals [48,49]. *g_Oscillibacter*, an anaerobic bacterium belonging to *p_Firmicutes*, has neither been purely cultured nor extensively investigated. In addition, it is sensitive to probiotics, heavy metals, and diets [50]. Numerous studies on gut microbiota homeostasis have reported an increased abundance of *g_Oscillibacter*, *g_Ruminiclostridium*_*9*, and *g_Lachnospiraceae_UCG-006* in animal models of UC or patients with UC [12,51]. Consistently, this study showed that treatment with DSS increased the abundance of the aforementioned bacteria, whereas treatment with LBE counteracted the effects of DSS. Altogether, these findings suggest that LBE alleviates gut microbiota dysbiosis in DSS-induced UC mice by restoring the abundance of *g_Akkermansia*, *g_Lactobacillus*, *g_Blautia*, *g_Lachnospiraceae_UCG-006*, *g_Oscillibacter*, *g_Erysipelatoclostridium*, and *g_Ruminiclostridium_9*.

## 4. Materials and Methods

### 4.1. Materials and Reagents

LB was purchased from Anguojiashuo Co., Ltd. (sample no. 202104, Anguo, China). DSS was purchased from MP Biomedicals, LLC (160110, Illkirch, France). A Griess reagent kit was obtained from Invitrogen, Thermo Fisher Scientific (G7921, Eugene, OH, USA). A mouse TNF-α kit (62MTNFAPEG), mouse IL-6 kit (62MIL06PEG), and mouse IL-1β kit (62MIL1BPEG) were purchased from Cisbio Bioassays (Codolet, France). The macrophage cell line RAW 264.7 was obtained from the American Type Culture Collection (TIB-71, VA, USA). A CCK-8 kit was purchased from Topscience Co. Ltd. (Shanghai, China). The Eastep^®^ Super Total RNA Extraction kit was obtained from Promega (Shanghai, China). ReverTra Ace qPCR RT Master Mix was purchased from TOYOBO Biotech Co., Ltd. (Shanghai, China). Platinum^®^ SYBR^®^ Green qPCR Supermix-UDG with ROX (C11744-500) was purchased from Invitrogen (Shanghai, China).

### 4.2. Preparation of LBE

A total of 100 g of dried LB whole plant powder was subjected to reflux extraction in 800 mL of 75% ethanol for 3 h. The resulting hot extract was directly filtered, and ethanol was removed through evaporation. Subsequently, the extract was lyophilized to yield LBE.

### 4.3. Cell Culture

RAW 264.7 macrophages were cultivated in Dulbecco’s modified Eagle medium (DMEM) supplemented with 10% fetal bovine serum (FBS) and 1% penicillin–streptomycin (P/S). The cells were preserved in a humidified incubator supplemented with 5% CO_2_, maintained at a constant temperature of 37 °C. When the cells reached 80–90% confluence after 3–4 days, they were sub-cultured in a split ratio of 1:3. Subsequent experiments were performed exclusively using cells from the logarithmic growth phase.

### 4.4. NO Inhibition Assay

RAW264.7 cells were seeded in 24-well plates at a density of 1 × 10^5^ cells/well and cultured in a humidified incubator with 5% CO_2_ at 37 °C for 12 h. After the cells had adhered to the culture plates, they were treated with 10–100 μg/mL concentrations of LBE or DMSO (vehicle) and LPS (1 μg/mL). After 24 h of incubation, the supernatant was aliquoted to assess nitrite concentration using the Griess reagent kit according to the manufacturer’s instructions. Briefly, the samples were incubated for 30 min with Griess reagent at room temperature, and optical density (OD) was measured at a wavelength of 548 nm using a microplate reader. Subsequently, the inhibition rate of NO production was calculated using the following formula:

Nitrite inhibition (%) = 100 − (OD_LBE_ − OD_vehicle_)/(OD_LPS_ − OD_vehicle_) × 100%

### 4.5. Cell Viability Assay

RAW 264.7 macrophages were seeded in a 24-well plate with 2 × 10^5^ cells per well and cultured in a humidified atmosphere comprising 5% CO_2_ at 37 °C for 24 h. Subsequently, 30 μL of CCK-8 reagent was introduced into each well, followed by incubation of the plate for approximately 1 h until the culture supernatant developed an orange color. The OD was then quantified at a wavelength of 450 nm utilizing a microplate reader, and the percentage of cell viability was calculated using the following formula:

Cell viability (%) = (OD_LBE_ − OD_blank_)/(OD_vehicle_ − OD_blank_) × 100%

### 4.6. Animals and Treatment

A total of 24 male C57BL/6J mice (age, 6–8 weeks; weight, 18–22 g) were obtained from Shanghai SLAC Laboratory Animal Co., Ltd. (Shanghai, China). All mice were maintained on a 12 h light/12 h dark cycle at a controlled temperature of 22 °C with a humidity level of 50% and had unrestricted access to food and water. All experiments procedures involving animals were executed in adherence to the directives established by the Association for Research at the Third Institute of Oceanography, as articulated in the ‘Statement for the Use of Animals in Pharmacological Research’, and received approval from the Experimental Animal Ethics Committee of the Third Institute of Oceanography, Ministry of Natural Resources (license number: TIO-IACUC-03-2021-03-01). The mice were randomly allotted into three distinct groups (n = 8) as follows: a control group, a model group, and an LBE-treated group. To induce acute colitis, mice in the model and LBE groups were administered 3% (*w*/*v*) DSS in drinking water for 7 days. Mice in the LBE group were intragastrically administered LBE, which was suspended in 0.1% carboxymethyl cellulose (CMC-Na) at a dose of 30 mg/kg of body weight for 7 days. The total disease activity index (DAI) score, ranging from 0 to 12, was calculated as the cumulative score of the following three criteria [11]: stool consistency (0 points for normal stools, 2 points for loose stools, 4 points for diarrhea), rectal bleeding (0 points for the absence of blood, 1 point for a positive hemoccult result, 2 points for visual pellet bleeding, and 4 points for gross bleeding), and body weight loss (0 points for no weight loss, 1 point for <5% weight loss, 2 points for 5–10% weight loss, 3 points for 10–20% weight loss, and 4 points for >20% weight loss). Mice were anesthetized with isoflurane, and blood samples were collected from the orbital venous plexus. Subsequently, the mice were sacrificed via cervical dislocation. Colon length was measured after the entire colon was harvested and washed with ice-cold PBS.

### 4.7. Macroscopic Scoring and Histochemical Analysis

Macroscopic scores were assigned based on the assessment of rectal bleeding (0 points for the absence of blood, 1 point for red blood, 2 points for dark red blood, and 3 points for gross bleeding), rectal prolapse (0 points for the absence of prolapse, 1 point for signs of prolapse, 2 points for clear prolapse, and 3 points for extensive prolapse), diarrhea (0 points for normal stools, 1 point for soft stools, 2 points for very soft stools, and 3 points for diarrhea), and colonic bleeding (0 points for a normal colon, 1 point for red blood, 2 points for dark red blood, and 3 points for black blood). After the colon was washed with ice-cold PBS and excess fluid was removed via blotting, approximately 5 mm of colon tissue was promptly fixed in 10% neutral-buffered formalin and subsequently sectioned into 5 μm thick sections. The sections were stained with hematoxylin and eosin (H&E) and photographed under light at a magnification of 200× (Nikon, Shanghai, China).

### 4.8. Detection of Pro-Inflammatory Cytokines in Serum

Whole blood samples from mice were centrifuged (3000 rpm, 10 min, 4 °C) to isolate serum. The concentration of pro-inflammatory cytokines, namely, TNF-α, IL-6, and IL-1β, in serum was measured using a mouse TNF-α kit, mouse IL-6 kit, and mouse IL-1β kit, respectively, according to the manufacturer’s instructions.

### 4.9. qRT-PCR

Total RNA was extracted from mouse colon tissues and purified using the Eastep^®^ Super Total RNA Extraction kit according to the manufacturer’s instructions. The extracted RNA was quantified on the NanoDrop 2000 spectrophotometer (Thermo Fisher Scientific, Waltham, MA, USA). A total of 1000 ng of RNA was reverse transcribed to cDNA using ReverTra Ace qPCR RT Master Mix according to the manufacturer’s instructions. Relative gene expression was quantified at the mRNA level using Platinum^®^ SYBR^®^ Green qPCR Supermix-UDG with ROX according to the manufacturer’s instructions. The 2^−ΔΔCt^ method was used to calculate the mRNA expression of TNF-α, IL-6, inducible nitric oxide synthase (iNOS), and cyclooxygenase-2 (COX-2), with GAPDH serving as the internal reference. The primer sequences used for PCR were synthesized by Sangon Biotech Co., Ltd. (Shanghai, China) and are listed in Table 3.

### 4.10. Identification of Major Chemical Components of LBE via LC-MS/MS

A total of 50 mg of lyophilized LBE was dissolved in 1.2 mL of 70% methanol. The mixture was vortexed for 30 s every 30 min six times and centrifuged at 12,000 rpm for 3 min. The supernatant was filtered using a membrane filter with 0.22 µm pore size (SCAA-104; ANPEL, Shanghai, China), and the resulting extract was analyzed via ultra-performance liquid chromatography (Nexera X2, SHIMADZU, Kyoto, Japan) coupled with electrospray ionization tandem mass spectrometry (QTRAP 4500, Applied Biosystems, MA, USA) (UPLC-ESI-MS/MS). The conditions for UPLC were as follows: column, Agilent SB-C18 (1.8 µm particle size, 2.1 mm × 100 mm); flow rate, 0.35 mL per minute; temperature, 40 °C; injection volume, 4 µL. The mobile phase gradient is specified in Table 4. The eluent was directed into the ESI-triple quadrupole-linear ion trap (QTRAP) MS system. The conditions for ESI were as follows: source temperature, 550 °C; ion spray voltage, +5500 V for positive ion mode and −4500 V for negative ion mode; ion source gases, GSI, GSII, and the curtain gas (CUR) used at 50, 60, and 25 psi, respectively. The collision-activated dissociation (CAD) voltage was set to high. For instrument tuning and mass calibration, 10 and 100 µM polypropylene glycol solutions were used in QQQ and LIT modes, respectively. QQQ scans were acquired through multiple reaction monitoring (MRM), with the collision gas (nitrogen) set to medium. The declustering potential (DP) and collision energy (CE) were optimized for individual MRM transitions. A specific set of MRM transitions was monitored for a specific period, which corresponded to the metabolites eluted during that period.

### 4.11. Network Pharmacology Analysis

The major chemical components of LBE identified via LC-MS/MS analysis were subjected to network pharmacological analysis. Subsequently, potential targets of LBE were identified using the Swiss Target Prediction Database (http://www.swisstargetprediction.ch/) (accessed on 29 November 2023) [52], GeneCards (http://www.genecards.org/) (accessed on 29 November 2023) [53], and other databases containing information on therapeutic targets for UC. Finally, a chemical compound–target–disease network was constructed using the Cytoscape software (version 3.2.1, National Institute of General Medical Sciences, Bethesda, MD, USA) [54]. This network was used to identify common genes between the targets of the active components of LBE- and UC-related targets. The primer sequences of five targets of LBE considered to be involved in the treatment of UC are shown in Table 5.

### 4.12. 16S rRNA Sequencing of Gut Microbiota

The gut microbiota sequencing was conducted by Gene Denovo Co., Ltd. (Guangzhou, China). Briefly, the extraction of microbial DNA from mouse fecal specimens was performed utilizing the HiPure Stool DNA Kit (Magen, Guangzhou, China). The V3–V4 hypervariable region of the 16S ribosomal RNA gene was amplified through polymerase chain reaction (PCR) under the following parameters: initial denaturation at 94 °C for 2 min, followed by 30 cycles of denaturation at 98 °C for 10 s, annealing at 62 °C for 30 s, extension at 68 °C for 30 s, and final extension at 68 °C for 5 min. The primer sequences used for PCR are as follows: 341F, CCTACGGGNGGCWGCAG; 806R, GGACTACHVGGGTATCTAAT. Each PCR run was performed in triplicate, with the reaction mixture (50 μL) containing 5 μL of 10 × KOD buffer, 5 μL of 2 mM dNTPs, 3 μL of 25 mM MgSO_4_, 1.5 μL of each primer (10 μM), 1 μL of KOD polymerase, and 100 ng of template DNA. The resulting amplicons were separated on 2% agarose gels and purified using the AxyPrep DNA Gel Extraction Kit (Axygen Biosciences, Union City, CA, USA) according to the manufacturer’s instructions. The purified amplicons were quantified using the ABI StepOnePlus Real-Time PCR System (Life Technologies, Foster City, CA, USA). Subsequently, the amplicons were pooled at equimolar concentrations and subjected to paired-end sequencing (PE250) on an Illumina platform according to the standard protocol. The resulting raw sequence reads were deposited in the NCBI Sequence Read Archive (SRA) database for further analysis and research purposes.

### 4.13. Statistical Analysis

Data analysis was conducted utilizing GraphPad Prism 8 software (San Diego, CA, USA) and expressed as the mean ± SEM. Bioinformatic analysis, including the assessment of taxonomy, species richness, and species diversity, and related statistical analysis were performed using the Omicsmart software. One-way ANOVA was used to analyze differences among groups. A *p*-value of <0.05 indicated statistically significant differences.

## 5. Conclusions

In conclusion, this study demonstrated that laboratory-extracted LBE had protective effects against UC in vitro and in vivo. LBE markedly decreased the levels of pro-inflammatory cytokines and DAI scores and reversed histopathological manifestations in mice with DSS-induced UC. Network pharmacology analysis and qRT-PCR revealed five potential target genes of LBE (*Ptgs2*, *F2*, *Plg*, *Pparγ*, and *Gpr35*) involved in the treatment of UC. Furthermore, LBE restored the abundance of *p_Proteobacteria* and probiotics such as *g_Lactobacillus* and *g_Blautia* in mice with DSS-induced UC. These findings may facilitate the development of LBE-based therapeutic strategies for UC. However, the exact active compounds of LBE that target the identified five genes and regulate the composition of gut microbiota, as well as their precise mechanisms of action, warrant further investigation.

## Figures and Tables

**Table 1:** Major chemical components of LBE.

No.	Ionization Model	Compounds	RT (min)	Relative Content
1	[M − H]^−^	3-O-galloyl-d-glucose	1.54	4.43 × 10^7^
2	[M − H]^−^	1-O-galloyl-β-d-glucose	1.6	4.52 × 10^7^
3	[M − H]^−^	6-O-galloyl-β-d-glucose	1.61	4.29 × 10^7^
4	[M + H]^+^	N-benzylmethylene isomethylamine	1.92	2.70 × 10^7^
5	[M + H]^+^	N-benzoyl-2-aminoethyl-β-d-glucopyranoside	1.99	3.10 × 10^7^
6	[M − H]^−^	2,3-di-O-galloyl-β-d-glucose	2.26	5.39 × 10^7^
7	[M − H]^−^	Methyl gallate	3.17	3.23 × 10^7^
8	[M + H]^+^	Quercetin-3-O-(2″-O-arabinosyl) rutinoside	3.43	3.08 × 10^7^
9	[M + H]^+^	Isotamarixin	3.54	2.83 × 10^7^
10	[M + H]^+^	Kaempferol-3-O-glucorhamnoside	3.92	2.30 × 10^7^
11	[M − H]^−^	Gallic acid ethyl ester	3.94	4.92 × 10^7^
12	[M + H]^+^	Kaempferol-3-O-sambubioside	3.96	4.81 × 10^7^
13	[M + H]^+^	Isorhoifolin	3.96	2.93 × 10^7^
14	[M + H]^+^	Rhoifolin	4.12	2.65 × 10^7^
15	[M + H]^+^	Aurantiamide acetate	7.62	2.84 × 10^7^

**Table 2:** LBE–UC-related common targets and their degree scores.

Gene	Name	Degree
PTGS2	Prostaglandin endoperoxide synthase 2	13
PLG	Plasminogen	9
PPAR-γ	Peroxisome proliferator-activated receptor gamma	6
F2	Coagulation factor II, thrombin	6
GPR35	G protein-coupled receptor 35	5
ABCB1	ATP-binding cassette subfamily B member 1	3
MPO	Myeloperoxidase	3
SYK	Spleen-associated tyrosine kinase	3
IL2	Interleukin-2	3
AKT1	Akt serine/threonine kinase 1	3
MET	Met proto-oncogene, receptor tyrosine kinase	3
MMP9	Matrix metallopeptidase 9	3
CCR1	C-C motif chemokine receptor 1	3
MMP3	Matrix metallopeptidase 3	3
TNF	Tumor necrosis factor	2
RELA	Rela proto-oncogene, NF-kB subunit	2
CHEK2	Checkpoint kinase 2	2
TLR9	Toll-like receptor 9	2

**Table 3:** Primer sequences from 5′ to 3′.

Gene	Forward Primers	Reverse Primers
GAPDH	GGTGAAGGTCGGTGTGAACG	CTCGCTCCTGGAAGATGGTG
TNF-α	AATGGCCTCCCTCTCATCAGTTCT	TGAGATAGCAAATCGGCTGACGGT
IL-6	AATTAAGCCTCCGACTTGTGAAG	CTTCCATCCAGTTGCCTTCTTG
iNOS	CCCGTCCACAGTATGTGAGGAT	CATTACCTAGAGCCGCCAGTGA

**Table 4:** Mobile phase for UPLC: solvent A contains pure water with 0.1% formic acid, and solvent B contains acetonitrile with 0.1% formic acid.

Time (min)	Solvent A (%)	Solvent B (%)
0	95	5
9	5	95
10	5	95
11	95	5
14	95	5

**Table 5:** Primer sequences of potential targets of LBE from 5′ to 3′.

Gene	Forward Primers	Reverse Primers
Ptgs2	TGCACTATGGTTACAAAAGCTGG	TCAGGAAGCTCCTTATTTCCCTT
PLG	TCCCAATGAGGGACTAGAAGAG	CGGATCTGTAGTGTAGCACCA
PPAR-γ	GCCCTTTGGTGACTTTATGGA	GCAGCAAGGTTGTCTTGGATG
F2	TCCGGTAGAACCAGGCTTTTC	GGGCAAACCAATCACAAACAC
GPR35	GGTCACCTCCCTGGTAGTG	AGCGGCAGGTAGAATCCCA

## Data Availability

The data presented in this study are available on request from the corresponding author.
